# Alignment between Published Performance Metrics and Clinical Reporting in sgNIPT: Implications from a Recent Case

**DOI:** 10.1055/a-2918-5666

**Published:** 2026-08-05

**Authors:** Laura G. Hendon

**Affiliations:** 1 Department of Pediatrics and Obstetrics and Gynecology 21693 The University of Mississippi Medical CenterJacksonMississippiUnited States

## 

I recently read the case report by Dr. Hogg 1 describing discordant results between two single-gene non-invasive prenatal testing (sgNIPT) platforms for cystic fibrosis in a known carrier–carrier couple with a prior affected child. In this case, one platform provided false reassurance by returning a “decreased risk” result, while a second sgNIPT platform returned a “high-risk” result that was eventually confirmed by diagnostic amniocentesis. As the author notes, this case emphasizes that sgNIPT is a screening tool and not a replacement for diagnostic genetic testing in pregnancy.

I am in agreement with some of the limitations of the existing validation studies that Dr. Hogg discusses in his case report, including the retrospective study design, limited outcome ascertainment on low-risk cases, and non-genetic outcomes. However, I would like to bring attention to an additional issue that is not often discussed when evaluating sgNIPT platforms. That is, the relationship between published performance metrics for sgNIPT platforms and the reporting criteria actually used in clinical practice. Specifically, the analytic frameworks used in published studies of the Unity Complete Fetal Risk screen (BillionToOne, Inc.) do not directly correspond to the multicategory risk reporting provided to clinicians.

In Dr. Hogg’s case, the a priori risk for cystic fibrosis in that pregnancy was one in four, given both parents were known carriers of the condition. The first sgNIPT assay reported a fetal risk result of 1 in 51 that was categorized clinically as “decreased risk.” What is confusing about this clinical result classification is that it differs from result classifications used in the published validation studies of this assay. In other words, different metrics are used in clinical reporting and validation studies.

**Fig. 1:**
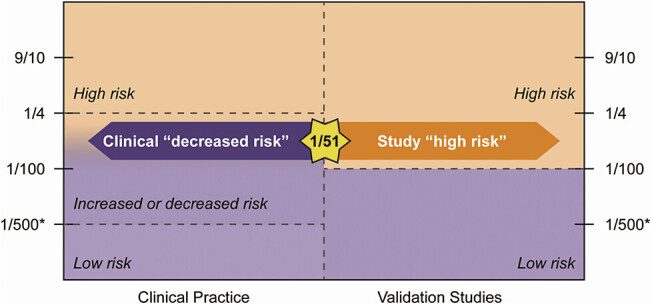
Clinical reporting and study categorizations. Illustration of how a single fetal risk estimate (1 in 51, as reported in Hogg’s case) is classified differently across published study frameworks and clinical reporting. 1 Differences in analytic thresholds across studies may complicate direct application of published performance metrics to clinical results. *About 1/400 used as a cut-off in Hoskovec et al.’s study, 2 1/500 used in Wynn et al.’s study, 3 and 1/4 used in Wynn et al.’s study. 4

In earlier validation studies, 2 3 test performance was evaluated using a binary classification system in which cases with estimated fetal risk greater than 1 in 100 were considered “high risk” and those at or below this threshold were considered “low risk” ( Fig. 1 ). Using this classification system, a result of 1 in 51 (like Dr. Hogg’s case) would be classified as “high risk” in this company’s validation studies. However, in clinical reporting, the same result is categorized as “decreased risk” within a four-category system (low risk, decreased risk, increased risk, high risk; Fig. 1 ).

Additional validation studies by this group 4 applied a different classification system using a less than one in four cut-off for “high-risk” classification, which changes how intermediate-risk results are ranked. Overall, these validation studies used multiple classification systems to evaluate the same sgNIPT assay, and none of these systems were the same as the four-category system used in this patient’s clinical report.

This discrepancy in classification systems could be confusing to clinicians. They might assume that the published sensitivity, specificity, and positive predictive value of the assay applies to the categories presented in patient reports. However, a single numerical risk estimate may be classified differently depending on the classification system used as “high risk” in one study, not high risk under another threshold, and “decreased risk” in the clinical report ( Fig. 1 ). As a result, performance metrics reported in the literature are not always directly translatable to the results clinicians receive and must interpret in practice.

The case presented by Hogg illustrates the challenge when different metrics are used in clinical reporting and validation studies. A result labeled “decreased risk” was returned in a clinical scenario with a one in four a priori risk, and the fetus was ultimately affected. Additional reports of false-negative results on this platform, including a recent group described by Zemanick et al., 5 further highlight the importance of understanding how test performance is described and communicated in the literature.

It is important to note that sgNIPT remains an extremely valuable screening tool, especially when couples at risk for single gene conditions do not or cannot pursue diagnostic genetic testing in pregnancy. However, it is important to remember that we need validation studies that evaluate test performance using the same categories and systems that are provided in clinical reports. As Hogg and others have noted, the best way to evaluate the accuracy of these assays is via prospectively designed studies that obtain genetic testing outcomes.

I agree with Dr. Hogg—diagnostic testing remains the gold standard for definitive prenatal diagnosis. At the same time, as sgNIPT continues to evolve and expand, clear alignment between published performance metrics and clinical reporting classification systems will be critical to support accurate interpretation, appropriate counseling, and informed clinical decision-making.
